# Cross-sectional surveillance of bluetongue virus, epizootic hemorrhagic disease virus, and chronic wasting disease in wild cervids in Nebraska

**DOI:** 10.3389/fvets.2026.1944700

**Published:** 2026-10-01

**Authors:** Duan S. Loy, Marnee K. Roundtree, Tanner Hurst, Joel Di Bernardo, Seth Lewin, Ahsan Naveed, Chelsea Lobb, Qin Lin, Colin Brewer, John Dustin Loy

**Affiliations:** 1 School of Veterinary Medicine and Biomedical Sciences, Nebraska Veterinary Diagnostic Center, University of Nebraska-Lincoln, Lincoln, NE, United States; 2 Nebraska Game and Parks Commission, Lincoln, NE, United States

**Keywords:** bluetongue virus, chronic wasting disease, *Culicoides*, epizootic hemorrhagic disease virus, spatial epidemiology, wildlife

## Abstract

Bluetongue virus (BTV) and epizootic hemorrhagic disease virus (EHDV) are *Culicoides*-transmitted orbiviruses, while chronic wasting disease (CWD) is an enzootic prion disease affecting North American cervids, primarily transmitted through contact with prions shed in bodily secretions and excretions. Recent work has shown the potential of retropharyngeal lymph nodes (RPLNs) for virus surveillance in addition to CWD and concurrent surveillance of these distinct diseases is limited. During the 2025 Nebraska hunting season, 780 RPLN samples were tested for CWD, BTV, and EHDV. Overall, 25.0% (195/780) were positive for CWD, followed by 17.9% (140/780) for BTV and 11.9% (93/780) for EHDV. Multivariable logistic regression identified higher odds of CWD detection in males than females [adjusted odds ratio (aOR) = 1.82; 95% CI: 1.12–2.96; *p* = 0.017] and lower odds in fawns than adults (aOR = 0.36; 95% CI: 0.21–0.62; *p* < 0.001). For BTV, fawns (aOR = 1.64; *p* = 0.042) and yearlings (aOR = 1.63; *p* = 0.032) had greater odds of detection than adults, whereas mule deer had lower odds than white-tailed deer (aOR = 0.34; *p* < 0.001). Mule deer also had lower odds of EHDV detection (aOR = 0.43; *p* = 0.013). Co-detection of two or more pathogens was identified in 9.9% (77/780) of animals, most commonly BTV and EHDV (4.2%; 33/780), whereas 1.4% (11/780) were positive for all three pathogens. Spatially, pathogen detection and co-detection rates varied across counties, with hotspots identified in southern Nebraska, particularly in Adams and Harlan counties. These findings demonstrate concurrent circulation and spatial clustering of prion and vector-borne viral pathogens, highlighting the need for integrated surveillance to better understand disease transmission dynamics in cervid populations.

## 1 Introduction

Infectious diseases play a critical role in shaping population dynamics, ecosystem health, and interactions at the wildlife, livestock, and human interfaces. White-tailed deer (WTD; *Odocoileus virginianus*) and mule deer (MD; *Odocoileus hemionus*) are two widely distributed cervid species in North America which are susceptible to a range of infectious diseases that pose ecological, agricultural, and potential public health concerns. Among these, bluetongue virus (BTV), epizootic hemorrhagic disease virus (EHDV), and chronic wasting disease (CWD) represent persistent and/or emerging threats in North American cervid populations (1–3). Declining North American cervid populations have been linked to outbreaks of hemorrhagic disease (HD) and enzootic circulation of CWD (4–8), but surveillance and scientific investigation into co-occurrence remain limited (9). Hemorrhagic disease (HD) reports in Great Plains states have increased throughout the years, yet Nebraska experienced reduced reporting of HD from 2012–2020 (10). Chronic wasting disease continues to spread both in Nebraska and beyond the Great Plains states (11). While Nebraska CWD surveillance is rotational around defined units, HD events are often observed and internally reported but not confirmed.

BTV and EHDV are closely related, segmented, double-stranded RNA (dsRNA) members of the genus *Orbivirus* (family *Sedoreoviridae*), transmitted by *Culicoides* spp. biting midges, and are responsible for seasonal outbreaks of HD in wild and domestic ruminants (12–14). White-tailed deer are particularly susceptible and may experience high morbidity and mortality during outbreaks, often serving as sentinels for virus activity (15, 16). The causal agents for the clinical manifestations of HD in wild cervids are indistinguishable between BTV and EHDV infections. Clinical signs include depression, weight loss, facial edema, fever, lameness, anorexia, reduced activity, and respiratory abnormalities (17, 18). Hemorrhagic disease is widely distributed across North America, with outbreaks typically occurring in late summer and early fall in association with peak vector activity and environmental conditions favoring midge abundance (19–21). In Nebraska, periodic outbreaks of HD have been documented (22, 23) but population-level estimates of viral exposure remain limited (14, 24, 25). One challenge for population-based assessments of viral exposure is sample collection. Recent studies have demonstrated the utility of retropharyngeal lymph nodes (RPLN) as a potentially useful sample type to determine population-level viral dynamics (26), as viral RNA can persist in lymph nodes. Therefore, this study evaluated the utility of RPLNs collected through routine CWD surveillance, in the absence of any reported mortality or disease events, for concurrent monitoring of hemorrhagic disease virus circulation.

CWD, a fatal transmissible spongiform encephalopathy caused by misfolded prion proteins, continues to expand its geographic range across North America (27, 28). The disease affects free-ranging and captive cervid populations, including white-tailed deer, mule deer, and elk, and is characterized by long incubation periods and persistent environmental transmission (29–31). CWD was first identified in Nebraska in 1998 in captive elk herds and confirmed in free-ranging deer in 2000. Deer Management Units (DMUs) are delineated to account for deer population diversity and variation and provide standardized areas for monitoring and managing populations and harvest. Continued spread prompted the Nebraska Game and Parks Commission (NGPC) to implement annual surveillance, resulting in eventual confirmed CWD presence in every DMU deer populations. Several of these management units were included in Nebraska’s rotational CWD surveillance program and were among the areas prioritized for surveillance in 2025. The highest reported percent positivity of CWD in wild cervids was 30.9% in the Plains DMU in Nebraska in 2022. The positivity rate in the Frenchman unit reached 26.44% in 2022, whereas in 2021, the rate was 7.2% in Buffalo, 16.92% in Republican, and 14.96% in Platte (32).

Surveillance of wildlife diseases often relies on cross-sectional study designs due to logistical constraints associated with tracking individual animals over time. Cross-sectional surveillance provides a snapshot of disease occurrence in a population during a defined period and is well suited for estimating prevalence or percent positivity, identifying geographic patterns, and informing management decisions (33, 34). In cervids, such approaches commonly utilize samples collected through hunter harvest, targeted culling, or opportunistic mortality investigations, allowing integration of disease monitoring into existing management frameworks (2, 35).

Despite the recognized importance of BTV, EHDV, and CWD to cervid populations in Nebraska and across North America, comprehensive assessments that examine these pathogens concurrently in wild cervid populations remain limited. The objective of this study was to evaluate positivity, geographic distribution, and co-detection patterns of BTV, EHDV, and CWD in wild cervids using RPLN testing across Nebraska. This will provide baseline information to inform wildlife disease management programs and future surveillance efforts.

## 2 Materials and methods

### 2.1 Study area

A cross-sectional study was conducted to determine the positivity rate of CWD, BTV, and EHDV in free-ranging white-tailed deer and mule deer in Nebraska. Samples were obtained from 32 counties. Most of these counties fall within four DMUs, including Buffalo, Republican, Platte, and Frenchman (Figure 1).

**Figure 1:**
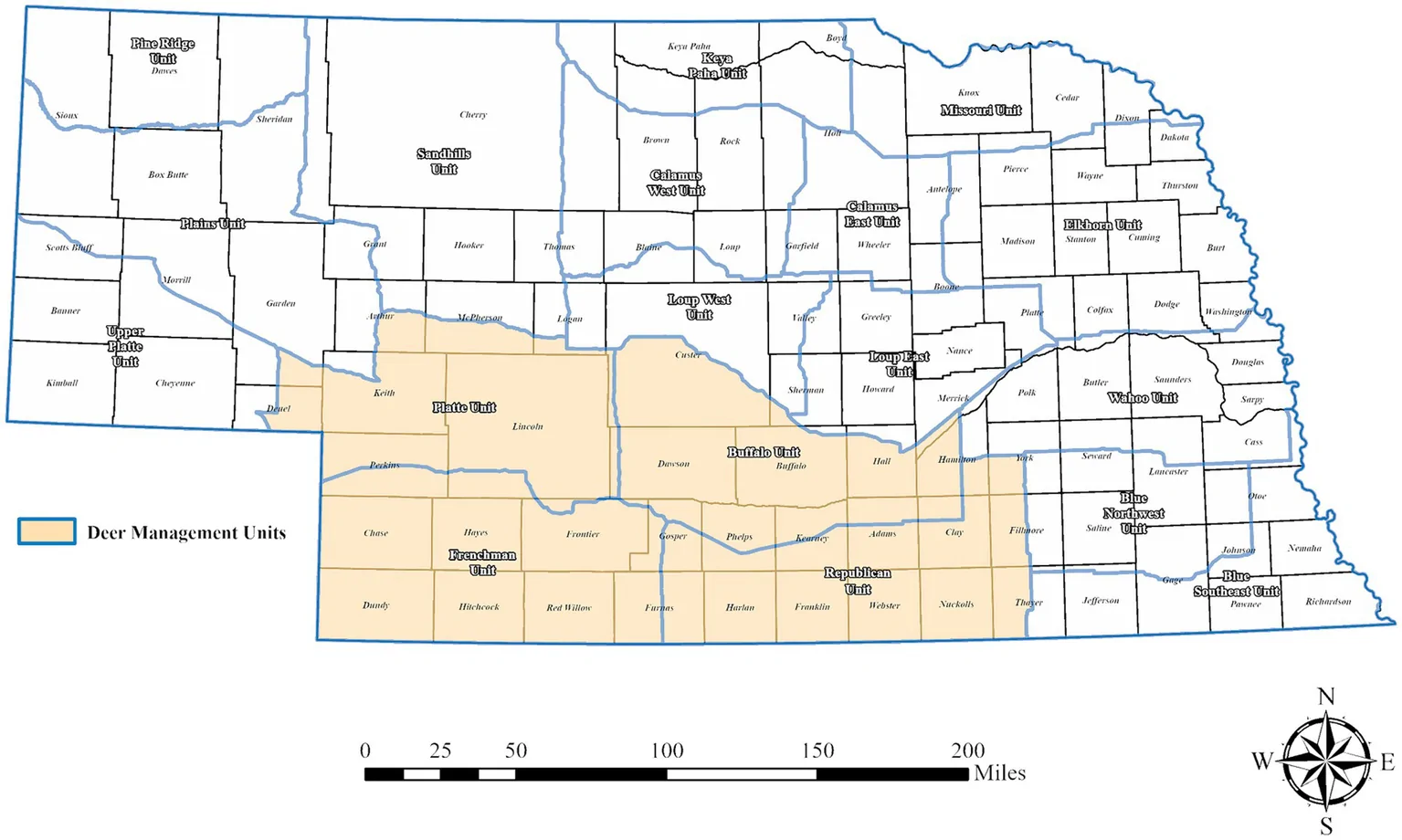
Spatial distribution of sampling regions. Samples were collected from two Deer Management Units (DMUs) in Southcentral Nebraska: Buffalo and Republican, and two DMUs in Southwest Nebraska: Platte and Frenchman.

### 2.2 Sample collection and storage

Samples were collected during annual wildlife disease surveillance activities. Samples were collected by NGPC staff at in-person check stations during the Nebraska firearm deer hunting season, and taxidermist sampling and individual hunters using self-sampling kits during Nebraska firearm and muzzleloader deer hunting seasons. A total of 780 white-tailed deer samples were available for testing. For each animal, RPLNs were collected, and tissues were placed into leak-proof containers and individually labeled with a unique identifier.

Sample-associated metadata were recorded for each sample, including date of collection, geographic location when available, sex, age class, and species. Sex was classified as male (M) or female (F). Age was assigned using standardized techniques based on tooth eruption and wear patterns (36). To summarize, fawns were categorized as deer <1 year, yearling >1 and <2 years, and adult >2 years. Species were recorded as white-tailed deer (WTD) or mule deer (MD).

Immediately following collection, samples were chilled in insulated coolers with ice packs. Samples were delivered or shipped to the Nebraska Veterinary Diagnostic Center (NVDC) on cold packs to preserve tissue integrity and pathogen viability. Upon receipt, samples were inventoried and stored at −20 °C or below prior to diagnostic testing in accordance with standard laboratory protocols.

### 2.3 Diagnostic testing

#### 2.3.1 ELISA testing for chronic wasting disease

RPLNs were tested for the presence of CWD antigen using a commercial enzyme-linked immunosorbent assay (ELISA) (HerdChek CWD Ag Test; IDEXX Laboratories, Westbrook, ME, USA), following the manufacturer’s protocol. In brief, 0.25–0.35 g of retropharyngeal lymph node tissue from right and left lymph nodes was placed in grinding tubes pre-filled with buffer and grinding beads. These were homogenized for 2 cycles of 30 s at 6500 rpm, with a 5 min rest between. Homogenates are then loaded into wells of an antigen capture plate and incubated. After a wash step, conditioning buffer is added, followed by the addition of a horseradish peroxidase conjugated anti-PrP antibody. After incubation and washing, TMB substrate is added and further incubated. The reaction is then stopped and the plate absorbance read at 450 nm using 650 nm reference wavelength. The cutoff value for samples is calculated according to manufacturer’s recommendation. Samples above the cutoff are considered positive and are subjected to immunohistochemistry (IHC) staining for confirmatory analysis. Samples yielding positive or suspect ELISA results were subsequently confirmed by IHC which was considered the reference confirmatory method for CWD diagnosis.

#### 2.3.2 Immunohistochemistry analysis of retropharyngeal lymph node samples

Detection of CWD prion protein by IHC was performed according to established protocols (37, 38). Briefly, RPLNs were collected as transverse sections. Tissues were fixed in 10% neutral buffered formalin (NBF) for a minimum of 24 h and embedded in paraffin wax. Paraffin-embedded tissues were sectioned at 4 μm, mounted on glass slides, dewaxed, and rehydrated through graded alcohols. Sections were then immersed in 95% formic acid for 5 min to enhance prion antigen detection, followed by multiple washes in Tris buffer (pH 7.5). Heat-induced antigen retrieval was performed using DIVA Decloaker solution (DV2004LX; BioCare Medical, Pacheco, CA) in a decloaking chamber. IHC labeling for the disease-associated prion protein (PrP^CWD^) was performed on the Ventana Discovery Ultra automated staining platform (Roche Diagnostics, Indianapolis, IN). The assay utilized the ready-to-use monoclonal antibody F99 (Anti-Prion Research Kit, Cat. No. 760–231; Roche Diagnostics, Indianapolis, IN), with detection via a Fast Red chromogen system (Activator Red, naphthol phosphate substrate) and hematoxylin counterstaining (Roche Diagnostics, Indianapolis, IN). Positive control tissues were included in each staining run to ensure assay performance. For lymphoid tissues, a minimum of six well-defined follicles was required for a sample to be considered adequate for interpretation as negative. Tissues were classified as positive when distinct, bright red, granular immunolabeling was observed within follicular germinal centers.

#### 2.3.3 Real-time PCR for BTV and EHDV

The same RPLN samples were tested for the presence of BTV and EHDV nucleic acid using a commercial multiplex real-time polymerase chain reaction (RT-qPCR) assay (RealPCR BTV/EHDV RNA Mix; IDEXX Laboratories, Westbrook, ME, USA). RPLNs were validated for use as a sample type for use in accordance with NVDC quality control system criteria. The assay was performed according to the manufacturer’s instructions. RPLN homogenate remaining after CWD ELISA testing was used for molecular analyses. Viral RNA was extracted from 300 μL of homogenate using the MagMAX™ Pathogen RNA/DNA Kit (Applied Biosystems, Thermo Fisher Scientific, Waltham, MA, USA) on a KingFisher Flex automated magnetic particle processor (Thermo Fisher Scientific) following the low cell protocol, with a final elution volume of 90 μL. Extracted RNA was heated at 95 °C for 3 min to denature double-stranded RNA (dsRNA) prior to molecular testing. RT-qPCR was performed using an Applied Biosystems QuantStudio™ 5 Real-Time PCR System (Thermo Fisher Scientific). Reverse transcription was conducted at 50 °C for 15 min, followed by denaturation at 95 °C for 1 min. Amplification consisted of 45 cycles of 95 °C for 15 s and 60 °C for 30 s, with fluorescence acquired at 60 °C. Each reaction included an internal positive control (IPC) to confirm assay performance. A cycle threshold (Ct) value ≤ 45 was considered positive. The Ct threshold for positivity (≤45) was based on the established performance characteristics of the validated diagnostic assay used at NVDC. Amplification curves were reviewed during result interpretation to ensure consistency with expected assay behavior. Results were linked to individual animal records using unique sample identifiers.

### 2.4 Data analysis

Associations between demographic characteristics and pathogen detection were evaluated using separate multivariable logistic regression models for CWD, BTV, and EHDV. For each model, pathogen detection status was treated as a binary outcome, and sex, age class, species, and DMU were included simultaneously as categorical explanatory variables. Females, adults, white-tailed deer, and Southcentral Nebraska (Buffalo and Republican) were used as the reference categories for sex, age class, species, and DMU, respectively. Adjusted odds ratios (aORs) and 95% confidence intervals (CIs) were calculated by exponentiating the estimated regression coefficients. Multivariable logistic regression analyses were performed in Python using the statsmodels package (version 0.14.6). Statistical significance was defined as P<0.05 . Animals with missing or unclassified information for sex, age class, species, or DMU were excluded using complete-case analysis.

A forest plot was created to visualize aORs and 95% CIs for associations between pathogen detection status and sex, age class, and species. Estimates were obtained from separate multivariable logistic regression models for CWD, BTV, and EHDV, with sex, age class, species, and DMU included simultaneously as covariates. The forest plot was generated and formatted for publication using Python with Matplotlib. Adjusted odds ratios were displayed on a logarithmic scale, with a reference line at aOR = 1. Pathogens were differentiated by symbol shape, and statistically significant associations were indicated by filled symbols and significance annotations (* P<0.05 ; ** P<0.01 ; *** P<0.001 ). Animals with missing or unclassified sex, age class, species, or DMU information were excluded from the multivariable analyses. Generative AI tools (Microsoft Copilot based on GPT-5) were used solely to assist with figure layout and graphic formatting. All statistical analyses, results, interpretations, and manuscript content were reviewed and verified by the authors.

### 2.5 Disease mapping

Geospatial analyses were performed in ArcGIS Pro version 3.4.5 (ESRI, Redlands, CA). Sample coordinates (latitude/longitude) were converted to point features using the “XY Table to Point” tool and attributed with diagnostic results for CWD, BTV and EHDV. Samples lacking coordinate data were excluded from point-based mapping to avoid introducing spatial bias. Notably, approximately 72 samples from Lincoln County, Nebraska contained county-level information, but no corresponding XY coordinates and were therefore omitted from spatial visualization (Supplementary Table 1). These samples were retained in all county-level positivity calculations and non-spatial analyses because county identifiers were available. Their exclusion affected only the spatial representation of detections and local density estimates in southwest Nebraska and did not influence county-level positivity rates or demographic analyses. Point maps were generated for single infection and co-infection, with symbology distinguishing between groups. Spatial clustering was assessed using heat map symbology, including separate visualizations for all positives. County boundaries and major water bodies were overlaid to provide geographic context, and inset maps highlighted areas of elevated case density. A reference map displaying county boundaries and NGPC deer management units for the 2025 deer hunting season was also created. Spatial data were created using imagery basemaps provided by Earthstar Geographics.

## 3 Results

### 3.1 Diagnostic test results

A total of 780 RPLN samples were submitted to the NVDC during the Nebraska hunting seasons from November 15th – December 31st, 2025, as part of annual CWD surveillance. Of these 780 samples, 195 were CWD-positive, resulting in a positivity rate of 25.0% (195/780). CWD was the most frequently detected pathogen among the sampled animals, followed by BTV (17.9%; 140/780) and EHDV (11.9%; 93/780). White-tailed deer accounted for 70.0% (546/780) of submissions, and males comprised 80.3% (626/780) of sampled animals. Adults were the most frequently sampled age class (46.2%; 360/780), followed by yearlings (31.4%; 245/780) and fawns (21.3%; 166/780) (Table 1). Nine animals had missing demographic information and were excluded from demographic subgroup analyses but remained included in the overall pathogen totals.

**Table 1:** Demographic characteristics and detection of chronic wasting disease (CWD), bluetongue virus (BTV), and epizootic hemorrhagic disease virus (EHDV) among 780 cervids sampled during the 2025 Nebraska hunting season.

Characteristic	Number of samples	Number of positives (%)		
		CWD ELISA	BTV RT-qPCR	EHDV RT-qPCR
Total sample	780	195 (25.0)	140 (17.9)	93 (11.9)
Species				
White-tailed deer	546	129 (23.6)	123 (22.5)	78 (14.3)
Mule deer	225	64 (28.4)	17 (7.6)	15 (6.7)
Age group				
Fawn	166	20 (12.0)	40 (24.1)	28 (16.9)
Yearling	245	74 (30.2)	49 (20.0)	27 (11.0)
Adult	360	99 (27.5)	51 (14.2)	38 (10.6)
Sex				
Male	626	169 (27.0)	106 (16.9)	70 (11.2)
Female	145	24 (16.6)	34 (23.4)	23 (15.9)

Nine animals had missing or unclassified species, age class, and sex information and were excluded from the corresponding demographic summaries. These animals remained included in the overall pathogen totals. Values are presented as number detected (% within category).

CWD ELISA-positive samples showed a mean optical density (OD) value of 3.88 ± 0.88, and those samples were confirmed by IHC. Representative IHC staining demonstrates prion protein deposition in RPLN (Supplementary Figure 1). All ELISA-positive samples were confirmed as CWD-positive by IHC (195/195), indicating complete concordance among the ELISA-positive samples evaluated. The mean RT-PCR cycle threshold (Ct) values for BTV and EHDV were 36.84 ± 3.81 and 37.38 ± 3.90, respectively.

Multivariable logistic regression models included 768 animals with complete information for sex, age class, species, and DMU (Figure 2). After adjustment for the other demographic and geographic covariates, males had greater odds of CWD detection than females (aOR = 1.82, 95% CI: 1.12–2.96; P=0.017 ), whereas fawns had lower odds of CWD detection than adults (aOR = 0.36, 95% CI: 0.21–0.62; P<0.001 ). CWD detection was not significantly associated with yearling age class or species after adjustment.

**Figure 2:**
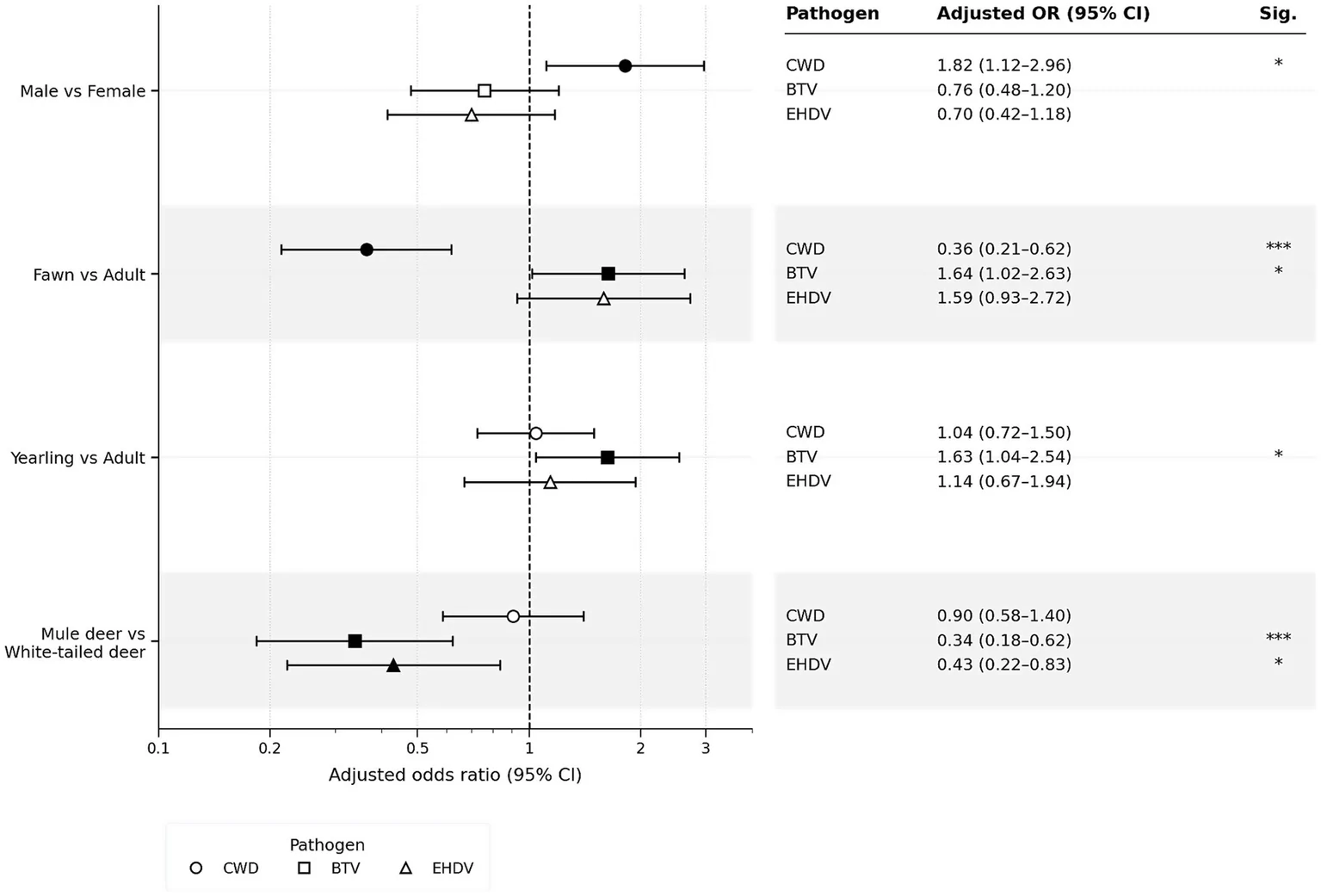
Forest plot showing adjusted odds ratios (aORs) and 95% confidence intervals (CIs) for associations between sex, age class, species, and detection of chronic wasting disease (CWD), bluetongue virus (BTV), and epizootic hemorrhagic disease virus (EHDV). Estimates were obtained from separate multivariable logistic regression models for each pathogen. Sex, age class, and species are shown in the figure, and all estimates were adjusted for Deer Management Units (DMU). Circles indicate CWD, squares indicate BTV, and triangles indicate EHDV. Filled symbols denote statistically significant associations ( P<0.05 ), whereas open symbols indicate non-significant associations. aORs are presented on a logarithmic scale, with the vertical dashed line representing aOR = 1. Statistical significance is indicated as follows: * P<0.05 ; ** P<0.01 ; *** P<0.001 .

Fawns (aOR = 1.64, 95% CI: 1.02–2.63; P=0.042 ) and yearlings (aOR = 1.63, 95% CI: 1.04–2.54; P=0.032 ) had greater odds of BTV detection than adults. Mule deer had lower odds of BTV detection than white-tailed deer (aOR = 0.34, 95% CI: 0.18–0.62; P<0.001 ). Sex was not significantly associated with BTV detection after adjustment.

Mule deer had lower odds of EHDV detection than white-tailed deer (aOR = 0.43, 95% CI: 0.22–0.83; P=0.013 ). Sex and age class were not significantly associated with EHDV detection after adjustment. DMU was not independently associated with CWD, BTV, or EHDV detection in the corresponding multivariable models. Adjusted odds ratios and 95% confidence intervals for all multivariable models are presented in Figure 2.

Co-detection analyses were conducted to examine concurrent detection of CWD, BTV, and EHDV. Single-pathogen detections predominated, including animals positive only for CWD (19.4%, 151/780), BTV (9.1%, 71/780), and EHDV (5.3%, 41/780). Among dual detections, BTV and EHDV co-detection was most common (4.2%, 33/780), followed by CWD and BTV (3.2%, 25/780) and CWD and EHDV (1.0%, 8/780). Eleven animals (1.4%, 11/780) were positive for all three pathogens (Supplementary Figure 2). Overall, 43.6% (340/780) of animals were positive for at least one pathogen, and 9.9% (77/780) were positive for two or more pathogens. These findings indicate concurrent circulation of CWD and hemorrhagic disease viruses within Nebraska cervid populations during the surveillance period.

### 3.2 Spatial distribution

At the county level, pathogen detection varied across the study region (Figure 3). Among counties with ≥10 samples, the CWD positivity rate ranged from 0 to 43.8% (7/16) in Kearney County, followed by 41.2% (7/17) in Chase County. The BTV positivity rate was highest in Harlan County at 56.0% (51/91), followed by 50.0% (7/14) in Adams County. The EHDV positivity rate was highest in Logan County at 50.0% (5/10), followed by Adams County at 35.7% (5/14) and Harlan County at 26.4% (24/91). Counties with small sample sizes (n < 10) exhibited a wide range of positivity rates (0–100%) (Supplementary Table 2).

**Figure 3:**
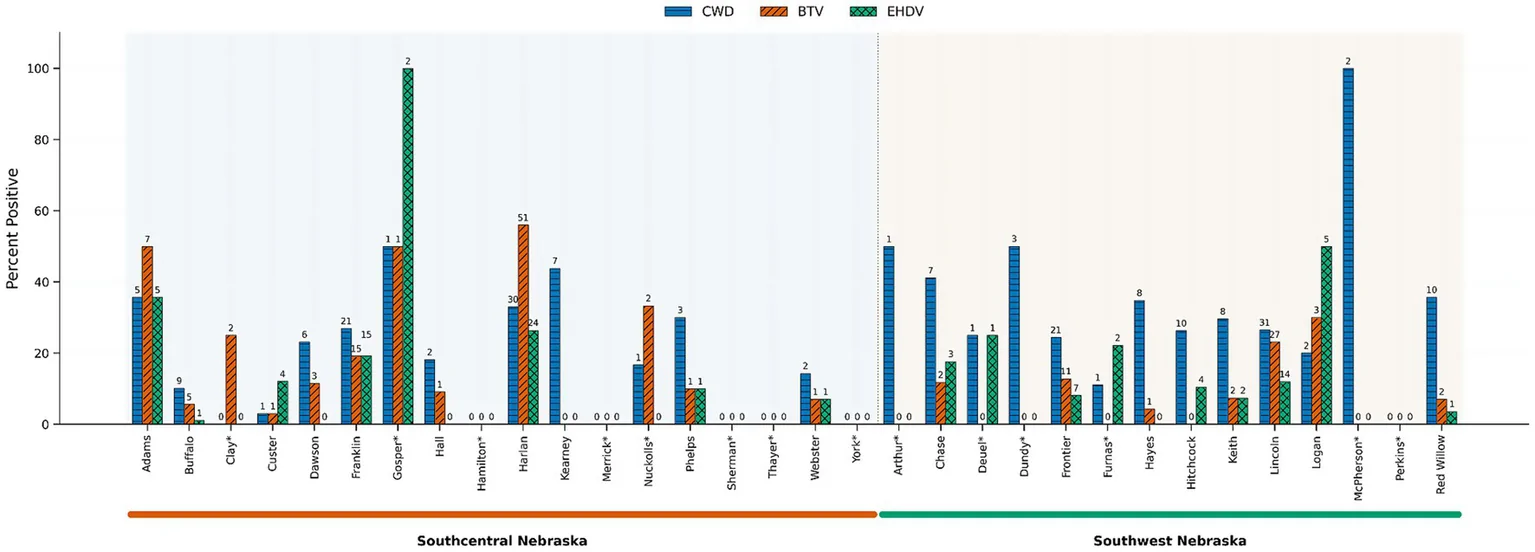
Percent positive for chronic wasting disease (CWD), bluetongue virus (BTV), and epizootic hemorrhagic disease virus (EHDV) by county within the Southcentral and Southwest Deer Management Units (DMUs) of Nebraska. An asterisk (*) indicates counties with fewer than 10 samples tested. Numbers above bars represent the number of positive samples for each pathogen.

Co-detection was identified in 9.9% (77/780) of samples. At the county level, co-detection positivity rates varied substantially, as shown in Supplementary Figure 3. The highest co-detection positivity rates were observed in Adams County (35.7%, 5/14), followed by Harlan County (31.9%, 29/91) and Logan County (30.0%, 3/10). Moderate co-detection positivity rates were observed in Franklin County (16.7%, 13/78), whereas lower rates were observed in Chase (11.8%, 2/17), Phelps (10%, 1/10), and Lincoln (9.4%, 11/117) counties. Most other counties exhibited low co-detection positivity (<10%). County-level hotspot analysis identified spatial clustering of co-detection as shown in Figure 4, with the strongest hotspots consistently observed in southern Nebraska counties including Adams and Harlan counties.

**Figure 4:**
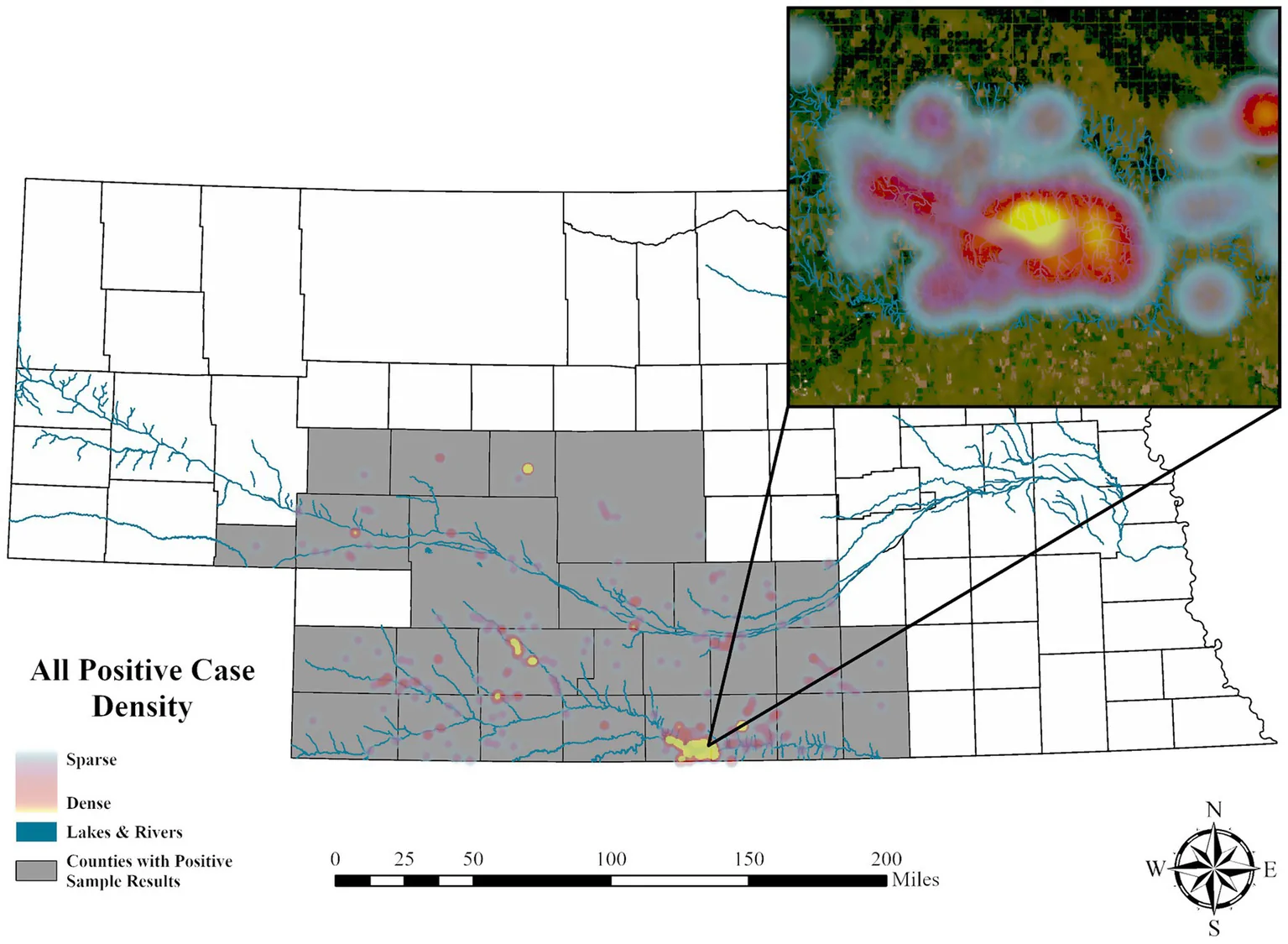
Spatial patterns of co-infected and clustered cases of chronic wasting disease (CWD), bluetongue virus (BTV), and epizootic hemorrhagic disease virus (EHDV) were identified using ArcGIS Pro version 3.4.5, with heat map symbology highlighting higher case density and major water bodies and county boundaries overlaid for interpretation; spatial data were generated using imagery basemaps from Earthstar Geographics.

## 4 Discussion

In this study, we describe the concurrent circulation of CWD and two vector-borne hemorrhagic disease viruses, BTV and EHDV, in Nebraska free-ranging cervid populations. CWD exhibited the highest positivity rate among the three tested diseases, consistent with its established enzootic status in the region. In contrast, BTV and EHDV were detected at lower, but notable frequencies, reflecting recent or concurrent circulation and transmission during the surveillance period. After adjustment for sex, age class, species, and DMU, CWD infection was associated with sex and age, with higher odds in males and lower odds in fawns, consistent with reports of male-biased prevalence in cervid populations (39, 40). However, because samples were derived from opportunistic hunter-harvested submissions rather than random population-based sampling, demographic biases may have influenced these associations. Male deer comprised 80.3% of submitted animals, and while males remained at increased odds of CWD detection after adjustment, this finding should not be interpreted solely as evidence of greater biological susceptibility, as hunter selectivity and other unmeasured sampling-related factors may also contribute. These patterns may reflect sex-specific behaviors, such as increased movement and contact rates, as well as the prolonged incubation period of prion diseases, resulting in cumulative infection risk with age (39, 41). Conversely, no species difference was observed for CWD after adjustment, indicating that CWD positivity was not associated with species in this study population. However, previous studies have reported greater CWD prevalence in mule deer than in white-tailed deer, particularly in western North American cervid populations, suggesting that species-associated risk may vary geographically (40, 42, 43).

BTV detection was associated with age class and species, but not sex after adjustment for the other demographic and geographic covariates. Fawns and yearlings had higher odds of BTV detection than adults. Although fawns also had higher estimated odds of EHDV detection than adults, this association was not statistically significant in the multivariable analysis. Mule deer had lower adjusted odds of BTV and EHDV detection compared to white-tailed deer, consistent with previous reports describing a greater burden of hemorrhagic disease in white-tailed deer (1, 29). These findings are consistent with vector-borne transmission driven by *Culicoides* midges, where exposure is shaped by environmental and ecological factors rather than host sex (44, 45). Additionally, younger animals may also be more likely to have more recent active virus infections, as compared to older animals who may have mounted an effective immune response in previous seasons. The contrasting patterns of CWD, BTV, and EHDV detection should be interpreted with caution, as the data were derived from surveillance submissions rather than from a systematically sampled population. Consequently, observed differences among age classes, sexes, and species may reflect both biological factors and potential biases in sample collection and submission practices.

Because the surveillance program was designed primarily around CWD sample collection, only RPLNs were available for BTV and EHDV testing. Although molecular testing was performed using a sensitive RT-qPCR assay with 45 amplification cycles, RPLNs may not be the optimal tissue for detection of these hemotropic viruses, which are frequently tested for in blood samples. Consequently, the reported BTV and EHDV positivity rates may underestimate true prevalence because low viral loads, postmortem tissue degradation, and the use of lymphoid tissue rather than whole blood or parenchymal organs could reduce diagnostic sensitivity and increase the likelihood of false-negative results. Future studies incorporating paired whole blood, spleen, and lung samples collected during acute disease investigations may provide more accurate estimates of BTV and EHDV prevalence (46, 47).

The observed CWD positivity rate aligns with prior surveillance indicating sustained transmission in southcentral and western Nebraska. Detection of CWD across multiple counties, including both historically enzootic and adjacent areas, suggests continued geographic persistence rather than localized introduction events (32). Counties with a higher positivity rate also tended to have greater sampling intensity, underscoring the importance of sustained surveillance for accurate spatial interpretation. While inference about true positivity rate is limited by the opportunistic sampling study design during hunting season, the spatial distribution of detections supports classifying CWD as enzootic across broad geographic regions of Nebraska.

BTV and EHDV detections were geographically widespread and occurred across multiple regions, reflecting patterns observed during sample collection in the late fall during deer hunting season in Nebraska, when environmental and climatic conditions support *Culicoides* vector activity and transmission dynamics (12, 48, 49). The detection of hemorrhagic disease viruses in counties without concurrent CWD detections highlights the independent transmission ecology of these agents and suggests that hemorrhagic disease viruses circulation is not restricted to areas with known enzootic CWD. A notable finding of this study was the widespread detection of BTV and EHDV RNA in RPLN from hunter-harvested cervids that did not have any history or suspected hemorrhagic disease. Previous prospective studies have shown that white-tailed deer may become infected and seroconvert to EHDV without being identified through mortality investigations, suggesting that subclinical or clinically unrecognized infections occur (50). Serologic surveillance data from other states, absent any reported disease, have shown 84% seropositivity for BTV and EHDV in some WTD populations, supporting the observation that many of these cases are non-clinical infections (16, 51). Additionally, given there are 15 or more serotypes of BTV and at least 3 types of EHDV present in the US, the potential for variation in potential pathogenicity and host susceptibility among circulating virus populations exists (12). Our findings support these observations that some infected animals survive infection and remain available for harvest, and that mortality-based surveillance may underestimate the geographic extent of hemorrhagic disease virus circulation. Nevertheless, because clinical histories were unavailable, asymptomatic infection could not be confirmed and these detections should be interpreted cautiously as evidence of potentially subclinical or clinically unrecognized infection.

This study also examined the presence of co-detections involving CWD and one or both hemorrhagic disease viruses. Dual infections were most common between BTV and EHDV, consistent with shared vector transmission pathways as previously reported (21, 30, 44). However, co-detections involving CWD and hemorrhagic disease viruses, including triple-positive animals, demonstrate that cervids may be exposed to and infected by both chronic prion diseases and acute viral pathogens within the same season. These findings should be interpreted cautiously. Detection of pathogen nucleic acid or antigen does not imply simultaneous clinical disease, nor does it establish causal interactions between pathogens. CWD is a chronic, slowly progressive prion disease, often persisting for months to years before clinical signs or mortality occur (28, 52). In contrast, BTV and EHDV are acute, vector-borne viral infections, typically leading to short infection windows, seasonal outbreaks, and in some cases rapid mortality (13, 53). Nonetheless, concurrent infections may have implications for host health, immune function, or survival that warrant further investigation. Prion diseases are known to interact with the host immune system in complex ways, including limited adaptive immune recognition and replication within lymphoid tissues, and may be influenced by co-infection or inflammatory states (54, 55). However, direct evidence for associations between CWD and episodic hemorrhagic disease outbreaks in cervids remains lacking.

Analyses revealed heterogeneity in co-detection patterns inter-regionally. Southcentral (Adams and Harlan counties) and southwest Nebraska (Lincoln and Frontier counties) exhibited the greatest variation of infection combinations, including multiple dual and triple co-detections, likely reflecting overlap between established CWD enzootic zones and seasonal BTV and EHDV transmission. The hotspot (Figure 4. inset maps) highlights a cluster of positive cases near Harlan County Reservoir, consistent with patterns reported in other states, such as Illinois, where hemorrhagic disease-positive counties were concentrated along the Illinois River (56). These patterns may also be influenced by animal aggregation behavior. During the hunting season, deer frequently congregate at shared water sources, such as reservoirs, which could promote environmental accumulation of prions and increase exposure to *Culicoides* vectors (50, 57, 58). Prion agents can persist in the environment and accumulate in areas frequented by cervids, whereas BTV and EHDV transmission occurs where competent vectors and susceptible hosts overlap spatially and temporally (45, 58). This represents a plausible and testable hypothesis linking host behavior, environmental contamination, and vector-mediated transmission.

Interpretation of the spatial analyses should also consider limitations associated with incomplete geographic data. Seventy-two samples from Lincoln County lacked geographic coordinates and therefore could not be included in point-based mapping or hotspot analyses. Although these samples were retained in county-level positivity estimates and all non-spatial analyses, their exclusion likely reduced point density within southwest Nebraska and may have underestimated local hotspot intensity in Lincoln County and surrounding areas. Consequently, spatial clustering patterns identified in southwest Nebraska should be interpreted conservatively, particularly when comparing hotspot intensity among regions.

The concurrent detection of CWD, BTV, and EHDV underscores the value of integrated wildlife disease surveillance. Surveillance programs often focus on individual pathogens; however, multi-pathogen approaches may provide a more complete understanding of wildlife health, disease ecology, and risk. Shared testing sample types also provide the ability to harmonize data collection and most cost effectively screen for additional disease agents. Identification of co-detection in this single year of surveillance provides preliminary information that may help guide future surveillance priorities, public outreach, and management decisions, particularly during years of elevated BTV and EHDV activity. Continued multi-year surveillance is needed to determine whether these patterns persist over time.

These findings highlight the complex pathogen landscape facing free-ranging cervid populations. They further demonstrate the utility of leveraging existing surveillance programs to simultaneously monitor multiple pathogens across large landscapes. Such approaches may provide a more comprehensive understanding of wildlife disease ecology and support evidence-based management decisions.

## Data Availability

The original contributions presented in the study are included in the article/Supplementary material, further inquiries can be directed to the corresponding author/s.
