# Supplementary material for: Cross-sectional surveillance of bluetongue virus, epizootic hemorrhagic disease virus, and chronic wasting disease in wild cervids in Nebraska
Source: Front Vet Sci. 2026 Oct 1;13:1944700. doi: 10.3389/fvets.2026.1944700 (PMC13668572; doi:10.3389/fvets.2026.1944700)
Supplement: Supplementary file 1 [file Data_Sheet_1.PDF]

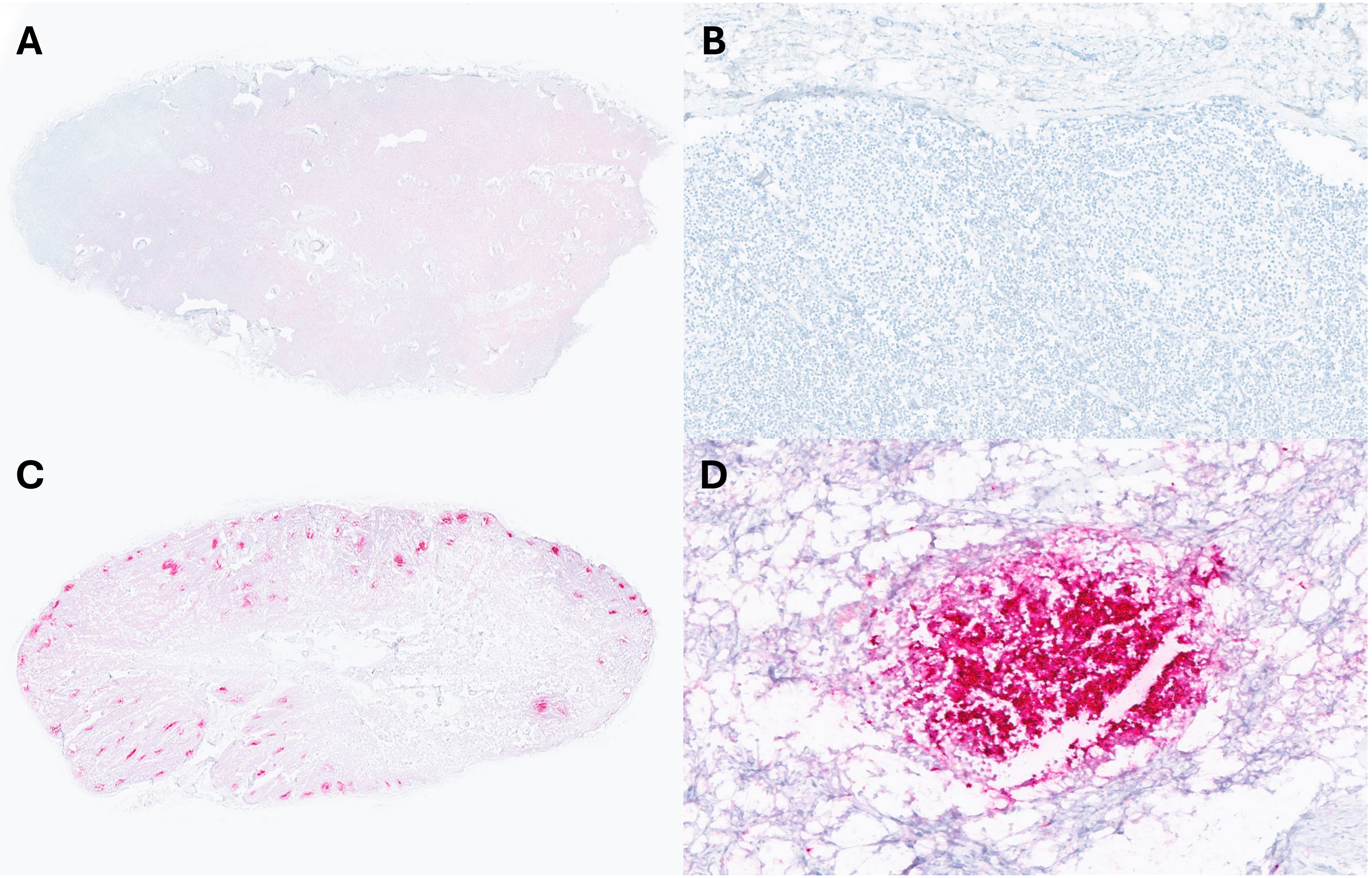

Supplementary Figure 1. Immunohistochemistry detection of PrP^{CWD} in retropharyngeal lymph node biopsies from white-tailed deer. Immunoreactivity for PrP^{CWD} is not detected in negative samples (A and B) and restricted to lymph node follicles in positive samples (C and D). Fast Red substrate assay with hematoxylin/bluing counterstain.
