# Supplementary material for: Cross-sectional surveillance of bluetongue virus, epizootic hemorrhagic disease virus, and chronic wasting disease in wild cervids in Nebraska
Source: Front Vet Sci. 2026 Oct 1;13:1944700. doi: 10.3389/fvets.2026.1944700 (PMC13668572; doi:10.3389/fvets.2026.1944700)
Supplement: Supplementary file 2 [file Data_Sheet_2.PDF]

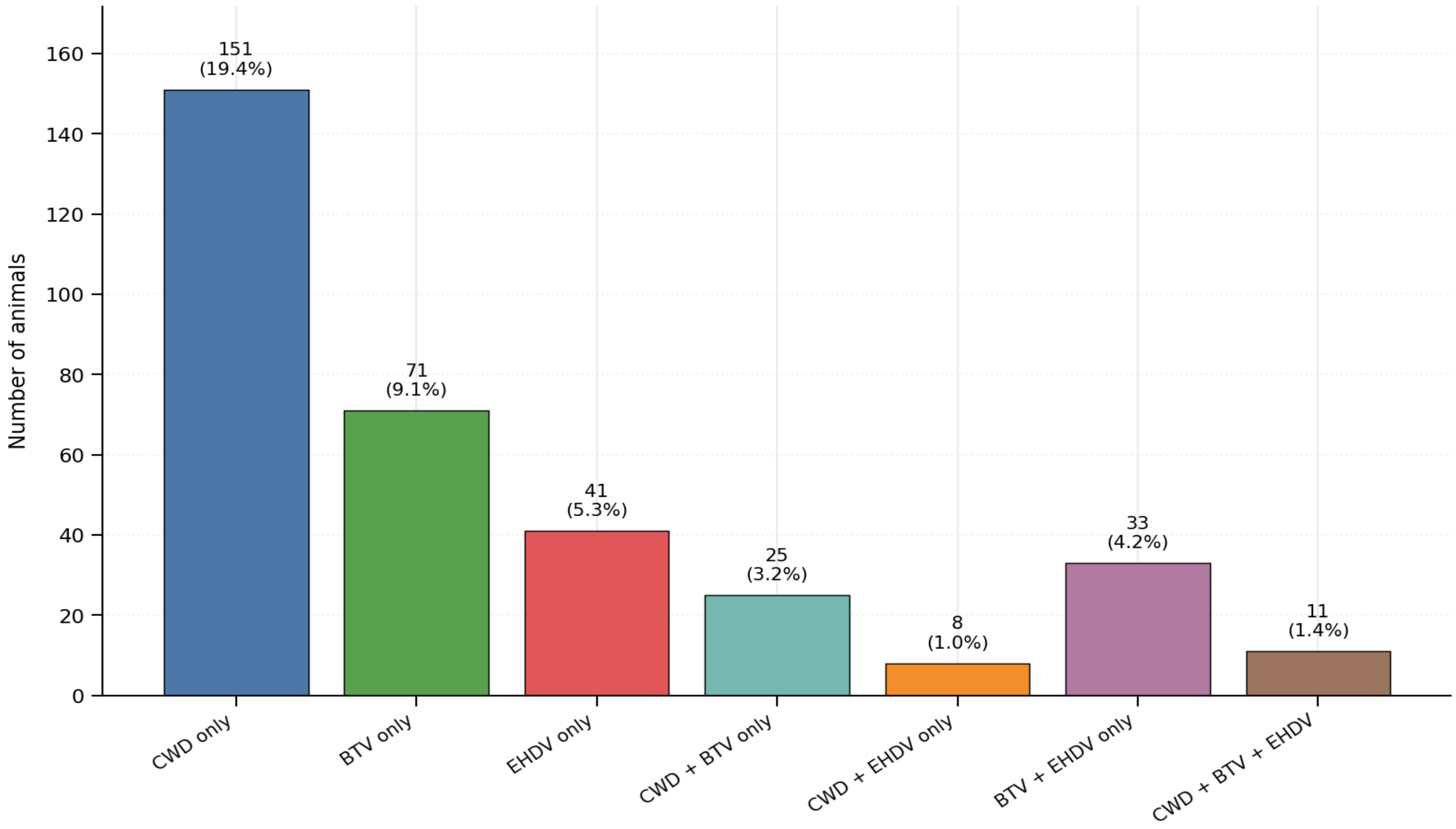

Supplementary Figure 2. Distribution of mutually exclusive single-pathogen detections and co-detections of chronic wasting disease (CWD), bluetongue virus (BTV), and epizootic hemorrhagic disease virus (EHDV) among cervids sampled during the 2025 Nebraska hunting season (n = 780). Counts and percentages are shown above the bars. Dual-detection categories exclude animals positive for all three pathogens; triple detections are presented separately.
