# Supplementary material for: Cross-sectional surveillance of bluetongue virus, epizootic hemorrhagic disease virus, and chronic wasting disease in wild cervids in Nebraska
Source: Front Vet Sci. 2026 Oct 1;13:1944700. doi: 10.3389/fvets.2026.1944700 (PMC13668572; doi:10.3389/fvets.2026.1944700)
Supplement: Supplementary file 3 [file Data_Sheet_3.PDF]

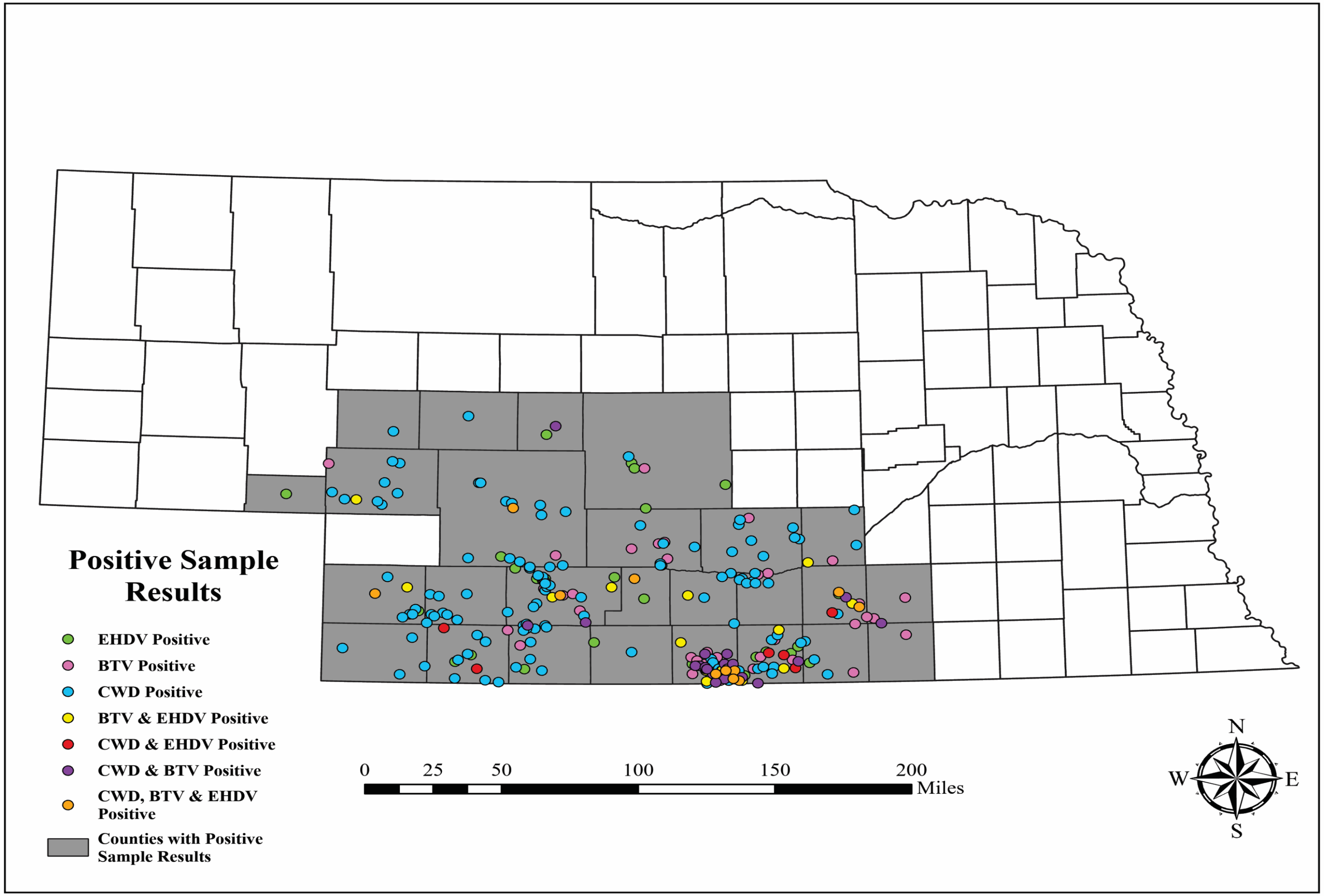

Supplementary Figure 3. Spatial distribution of samples positive for chronic wasting disease (CWD), bluetongue virus (BTV), and epizootic hemorrhagic disease virus (EHDV) for single and co-infections generated in ArcGIS Pro version 3.4.5 from georeferenced coordinates. Points are symbolized by infection status (single and co-infection), with county boundaries shown for geographic context.
