# Supplementary material for: Cross-sectional surveillance of bluetongue virus, epizootic hemorrhagic disease virus, and chronic wasting disease in wild cervids in Nebraska
Source: Front Vet Sci. 2026 Oct 1;13:1944700. doi: 10.3389/fvets.2026.1944700 (PMC13668572; doi:10.3389/fvets.2026.1944700)
Supplement: Supplementary file 4 [file Table_2.DOCX]

**Supplementary Table 2.** County-level detection of chronic wasting disease (CWD), bluetongue virus (BTV), and epizootic hemorrhagic disease virus (EHDV) among 780 cervids sampled from 32 counties during the 2025 Nebraska hunting season.

|  | Number of Samples | Number of Positives (%) | | |
| --- | --- | --- | --- | --- |
|  |  | **CWD ELISA** | **BTV RT-qPCR** | **EHDV RT-qPCR** |
| **County** |  |  |  |  |
| Adams | 14 | 5 (35.7) | 7 (50.0) | 5 (35.7) |
| Arthur | 2 | 1 (50.0) | 0 (0.0) | 0 (0.0) |
| Buffalo | 89 | 9 (10.1) | 5 (5.6) | 1 (1.1) |
| Chase | 17 | 7 (41.2) | 2 (11.8) | 3 (17.6) |
| Clay | 8 | 0 (0.0) | 2 (25.0) | 0 (0.0) |
| Custer | 33 | 1 (3.0) | 1 (3.0) | 4 (12.1) |
| Dawson | 26 | 6 (23.1) | 3 (11.5) | 0 (0.0) |
| Deuel | 4 | 1 (25.0) | 0 (0.0) | 1 (25.0) |
| Dundy | 6 | 3 (50.0) | 0 (0.0) | 0 (0.0) |
| Franklin | 78 | 21 (26.9) | 15 (19.2) | 15 (19.2) |
| Frontier | 86 | 21 (24.4) | 11 (12.8) | 7 (8.1) |
| Furnas | 9 | 1 (11.1) | 0 (0.0) | 2 (22.2) |
| Gosper | 2 | 1 (50.0) | 1 (50.0) | 2 (100.0) |
| Hall | 11 | 2 (18.2) | 1 (9.1) | 0 (0.0) |
| Hamilton | 1 | 0 (0.0) | 0 (0.0) | 0 (0.0) |
| Harlan | 91 | 30 (33.0) | 51 (56.0) | 24 (26.4) |
| Hayes | 23 | 8 (34.8) | 1 (4.3) | 0 (0.0) |
| Hitchcock | 38 | 10 (26.3) | 0 (0.0) | 4 (10.5) |
| Kearney | 16 | 7 (43.8) | 0 (0.0) | 0 (0.0) |
| Keith | 27 | 8 (29.6) | 2 (7.4) | 2 (7.4) |
| Lincoln | 117 | 31 (26.5) | 27 (23.1) | 14 (12.0) |
| Logan | 10 | 2 (20.0) | 3 (30.0) | 5 (50.0) |
| McPherson | 2 | 2 (100.0) | 0 (0.0) | 0 (0.0) |
| Merrick | 1 | 0 (0.0) | 0 (0.0) | 0 (0.0) |
| Nuckolls | 6 | 1 (16.7) | 2 (33.3) | 0 (0.0) |
| Perkins | 3 | 0 (0.0) | 0 (0.0) | 0 (0.0) |
| Phelps | 10 | 3 (30.0) | 1 (10.0) | 1 (10.0) |
| Red Willow | 28 | 10 (35.7) | 2 (7.1) | 1 (3.6) |
| Sherman | 1 | 0 (0.0) | 0 (0.0) | 0 (0.0) |
| Thayer | 1 | 0 (0.0) | 0 (0.0) | 0 (0.0) |
| Webster | 14 | 2 (14.3) | 1 (7.1) | 1 (7.1) |
| York | 1 | 0 (0.0) | 0 (0.0) | 0 (0.0) |
| Not Provided | 5 | 2 (40.0) | 2 (40.0) | 1 (20.0) |

*Note.* Values are presented as number detected (% within county). County-level positivity estimates include all samples with county information available, regardless of whether geographic coordinates were available for point-based mapping. Counties with fewer than 10 samples should be interpreted cautiously. Five samples lacked county information and are presented as “Not provided.”
